# Hydrogen Sulfide Protects Hyperhomocysteinemia-Induced Renal Damage by Modulation of Caveolin and eNOS Interaction

**DOI:** 10.1038/s41598-018-38467-6

**Published:** 2019-02-18

**Authors:** Sathnur Pushpakumar, Sourav Kundu, Utpal Sen

**Affiliations:** 1 Department of Physiology, School of Medicine, University of Louisville, Louisville, KY 40292 USA; 2 Department of Botany, West Bengal State University, Berunanpukuria, Kolkata, West Bengal PIN 700126 India

## Abstract

The accumulation of homocysteine (Hcy) during chronic kidney failure (CKD) can exert toxic effects on the glomeruli and tubulo-interstitial region. Among the potential mechanisms, the formation of highly reactive metabolite, Hcy thiolactone, is known to modify proteins by N-homocysteinylation, leading to protein degradation, stress and impaired function. Previous studies documented impaired nitric oxide production and altered caveolin expression in hyperhomocysteinemia (HHcy), leading to endothelial dysfunction. The aim of this study was to determine whether Hhcy homocysteinylates endothelial nitric oxide synthase (eNOS) and alters caveolin-1 expression to decrease nitric oxide bioavailability, causing hypertension and renal dysfunction. We also examined whether hydrogen sulfide (H_2_S) could dehomocysteinylate eNOS to protect the kidney. WT and Cystathionine β-Synthase deficient (CBS+/−) mice representing HHcy were treated without or with sodium hydrogen sulfide (NaHS), a H_2_S donor (30 µM), in drinking water for 8 weeks. Hhcy mice (CBS+/−) showed low levels of plasma H_2_S, elevated systolic blood pressure (SBP) and renal dysfunction. H_2_S treatment reduced SBP and improved renal function. Hhcy was associated with homocysteinylation of eNOS, reduced enzyme activity and upregulation of caveolin-1 expression. Further, Hhcy increased extracellular matrix (ECM) protein deposition and disruption of gap junction proteins, connexins. H_2_S treatment reversed the changes above and transfection of triple genes producing H_2_S (CBS, CSE and 3MST) showed reduction of vascular smooth muscle cell proliferation. We conclude that during Hhcy, homocysteinylation of eNOS and disruption of caveolin-mediated regulation leads to ECM remodeling and hypertension, and H_2_S treatment attenuates renovascular damage.

## Introduction

Hyperhomocysteinemia (Hhcy) is frequently seen in patients with chronic kidney disease (CKD) and recent studies implicate Hhcy in the pathophysiology of glomerulosclerosis and interstitial fibrosis, leading to progressive decline in function^1,2^. Hhcy causes arteriolar constriction, arterial stiffness and endothelial damage^3,4^. Impaired vascular response during Hhcy is attributed to decreased bioavailability of nitric oxide (NO). In the vasculature, NO is produced from L-arginine mainly by endothelial nitric oxide synthase (eNOS). Hhcy signals the formation of Hcy thiolactone and protein modifications known as homocysteinylation that damage proteins resulting in decreased biological activity. However, it is not known whether homocysteinylation of eNOS occurs during Hhcy.

The generation of NO is dependent on diverse agonists that activate eNOS. In the endothelial cells, eNOS is associated with special flask-shaped invaginations of plasmalemma of terminally differentiated cells called caveolae. Caveolin is a membrane protein in the caveolae which acts as a scaffold for proteins and lipids^5^. Three types of caveolin (Cav) have been described. Caveolin-1 and -2 are widely expressed in several tissues including kidney^6–8^ whereas caveloin-3 is exclusive to myocytes^9^. eNOS is a Ca^2+^/calmodulin dependent enzyme and its activity is regulated by its interaction with caveolin. When eNOS is bound to caveolin-1, the enzyme activity is attenuated whereas its dissociation from caveolin-1 increases enzyme function^5^. The effect of Hhcy on the expression and interaction between caveolin-1 and eNOS remains unknown.

In the body, homocysteine is metabolized mainly by the enzymes, cystathionine-β-synthase, cystathionine-γ-lyase, and 3-mercaptopyruvate sulfurtransferase (CBS, CSE and 3-MST respectively). This transsulfuration pathway yields cysteine and hydrogen sulfide (H_2_S). H_2_S is a gasotransmitter known to have multiple functions including regulation of vascular tone, neuromodulation, anti-oxidant and as an anti-inflammatory molecule^10,11^. A reduction of H_2_S producing enzymes and thus H_2_S has been implicated in animal model of CKD and clinical study^12,13^. Low plasma H_2_S has also been linked to decreased glomerular function and increased cardiac risk in CKD patients^14^. In contrast, supplementation of H_2_S has been shown to be beneficial in several studies^15–18^. Some of the beneficial effects of H_2_S are attributed to the activation of *K*_ATP_ (ATP-sensitive K+) channels or scavenging free radicals^19^. Recently, it has been speculated whether H_2_S is involved in posttranslational protein modification for some of its biological effects^20^. It is possible that H_2_S may modify the course of protein-S-S bridge formation and reverse homocysteinylation of proteins such as eNOS.

The aim of the present study was to investigate whether homocysteinylation of eNOS and disruption of caveolin-mediated eNOS regulation leads to hypertension and renal dysfunction. Further, we investigated whether H_2_S supplementation dehomocysteinylates eNOS and reduces vascular smooth muscle cell proliferation and extracellular matrix protein deposition to protect the kidney from Hhcy mediated injury.

## Results

### Plasma H_2_S, renal perfusion and glomerular filtration rate (GFR) is reduced during hyperhomocysteinemia

**Figure 1:**
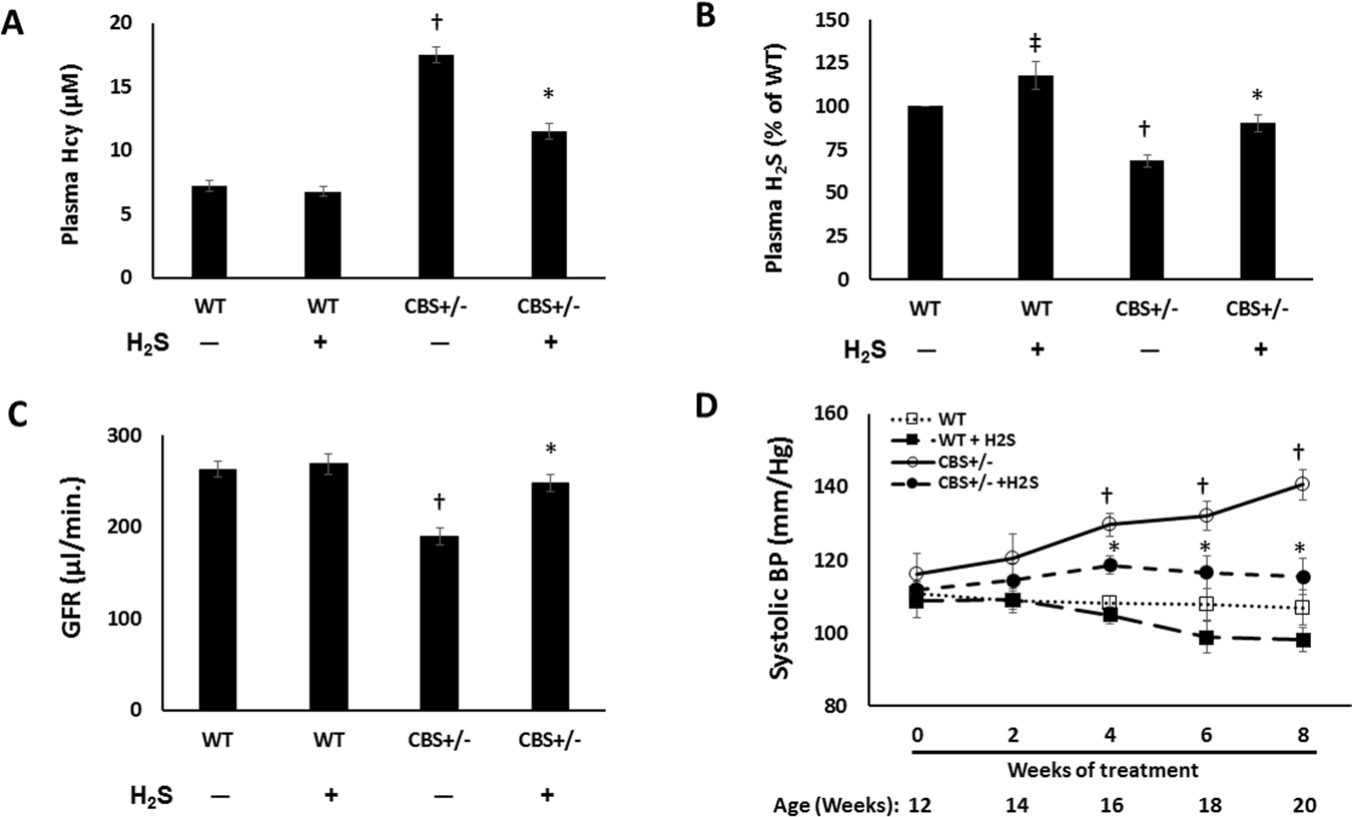
Hhcy decreases plasma H_2_S level and glomerular filtration rate (GFR) and H_2_S treatment improves GFR. Plasma Hcy was measured by HPLC and GFR by FITC Inulin method. (**A**) Plasma Hcy levels, (**B**) Plasma H_2_S levels and (**C**) Glomerular filtration rate (GFR), (**D**) H_2_S reduces systolic BP in hyperhomocysteinemic (CBS+/−) mice. Values are mean ± SEM, n = 7/group. *p < 0.05 vs. CBS+/− mice without H_2_S treatment, ^†^p < 0.05 vs. WT groups, ^‡^p < 0.05 vs. WT (control).

Plasma Hcy was measured by HPLC (Fig. 1A). Plasma Hcy was increased more than two-fold in CBS+/− mice compared to WT mice (Fig. 1A). In addition, CBS+/− mice had low levels of plasma H_2_S compared to WT groups (Fig. 1B). NaHS supplementation reduced plasma Hcy levels in CBS+/− mice and increased plasma H_2_S levels (Fig. 1A,B respectively). Plasma H_2_S increased in WT mice treated with NaHS (Fig. 1B).

FITC-Inulin clearance showed reduction of GFR in CBS+/− mice compared to WT groups without or with H_2_S treatment (Fig. 1C). Following H_2_S treatment, GFR returned to normal in CBS+/− mice suggesting recovery of renal function (Fig. 1C). GFR was not affected by H_2_S in WT mice.

At 12 weeks of age, there was no difference in systolic blood pressure (SBP) between the groups. CBS+/− mice exhibited progressive increase in SBP commencing at 16 weeks until the end-point of the experiment (20 wks) compared to WT mice (Fig. 1D). SBP was attenuated following H_2_S treatment in CBS+/− mice (Fig. 1D). There was no difference in the baseline SBP in WT and CBS+/− mice. H_2_S treatment did not affect the SBP in WT mice.

**Figure 2:**
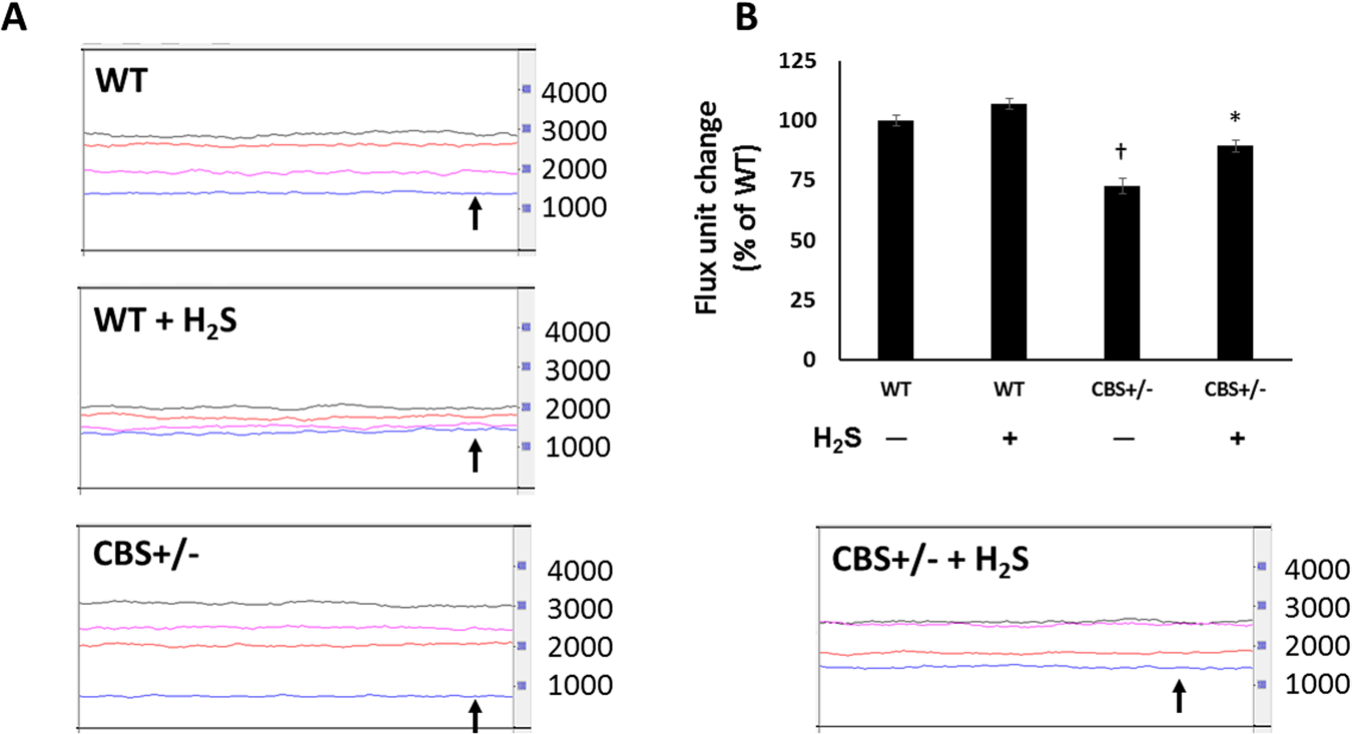
Hhcy reduces renal cortical blood flow. (**A**) Laser Doppler flowmetry line trace showing renal blood flow (blue line, black arrow) in the kidney. Black trace, is from aorta, red trace from renal artery and pink trace from renal vein. (**B**) Flux unit change from WT. Flux units = No. of RBCs x velocity. It is used as a surrogate for blood flow. n = 6/group, *p < 0.05 vs. CBS+/− mice without H_2_S treatment, ^†^p < 0.05 vs. WT groups.

Laser Doppler flowmetry was used to measure red blood cell flux (No. of RBCs × velocity) as an index of microvascular blood flow in the renal cortex. CBS+/− mice showed lower renal flux units compared to WT groups reflecting reduced perfusion (Fig. 2A, black arrow). H_2_S supplementation restored normal perfusion in CBS+/− mice (Fig. 2A,B). There was no difference renal flux units in WT groups without or with H_2_S treatment (Fig. 2A,B).

**Figure 3:**
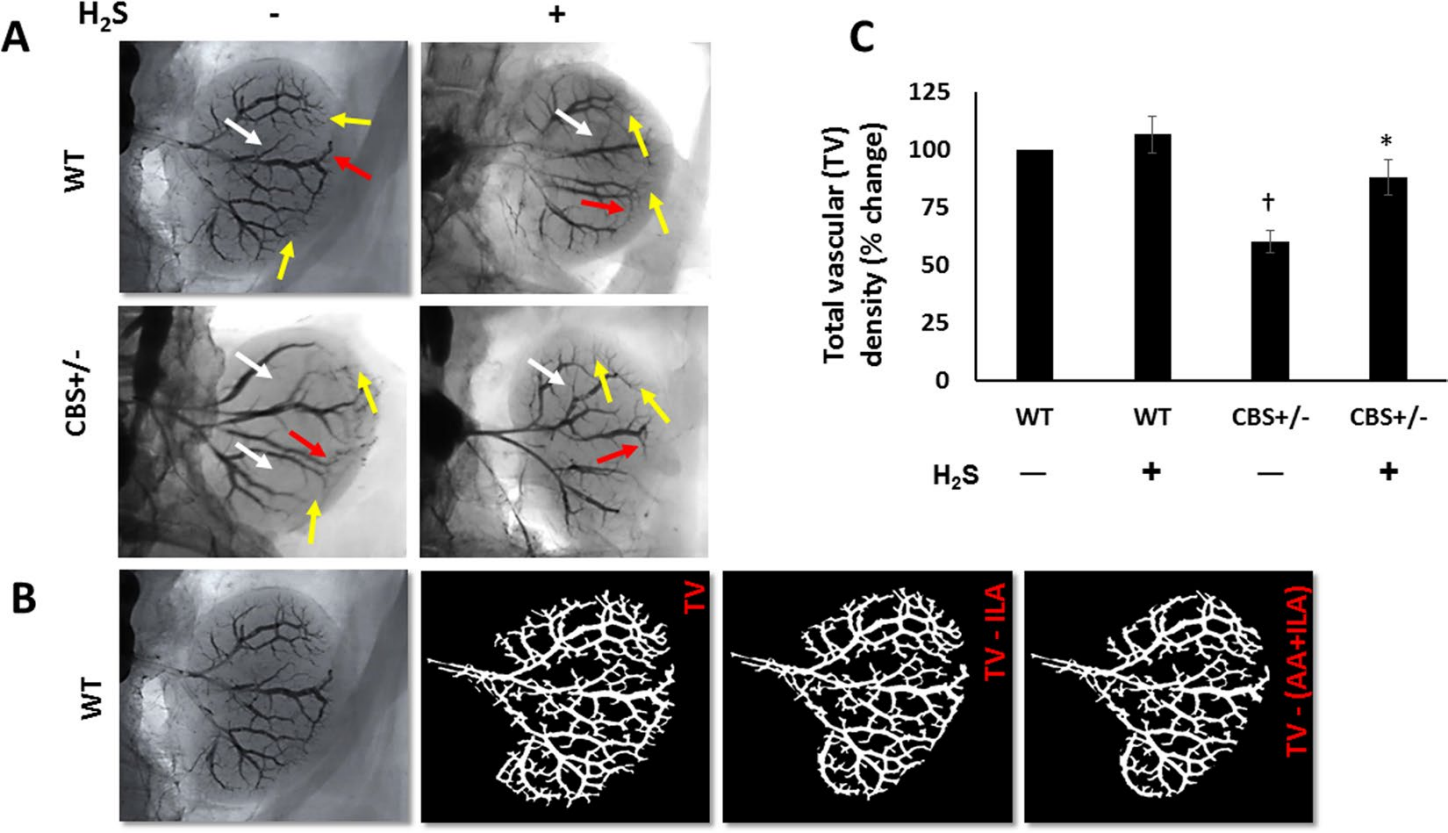
H_2_S restores Hhcy induced reduced renal cortical vascularity. (**A**) Representative barium angiogram of left kidneys showing total vascularity. Interlobar arteries are shown by white arrows, arcuate arteries by red arrows and interlobular arteries are shown by yellow arrows, (**B**) Analysis of renal vessels by Vessel Segmentation and analysis software, (**C**) Change in total vascular density as % from control mice (WT). n = 6/group, *p < 0.05 vs. CBS+/− mice without H_2_S treatment, ^†^p < 0.05 vs. WT groups. TV, total vascularity; ILA, interlobular artery; AA, arcuate artery.

Barium angiography revealed decreased vascular density in CBS+/− animals (Fig. 3A). In the renal cortex, CBS+/− mice demonstrated reduction of arcuate (Fig. 3A, red arrows) and interlobular arteries (Fig. 3A, yellow arrows) and the medulla showed decreased interlobar branches (Fig. 3A, white arrows). H_2_S enhanced vascular density by increasing all branches in the cortex and medulla (Fig. 3A,C) suggesting improved perfusion.

### Hhcy homocysteinylates eNOS, increases caveolin-1 expression and reduces nitric oxide production

**Figure 4:**
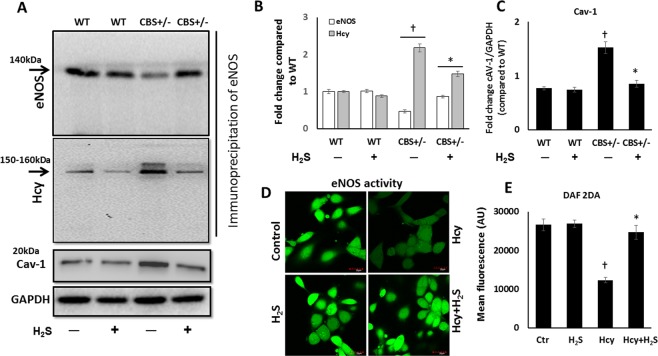
H_2_S increases eNOS activity by caveolin-1 modulation. (**A**) Immunoprecipitation of endothelial nitric oxide synthase (eNOS, conc.: 2 µg/200 µg of protein) and immunoblotting for eNOS (MW: 140 kDa) and Hcy (MW: 150–160 kDa) in the kidney. Caveolin-1 (Cav-1), (MW: 20 kDa) was quantified in the non-immunoprecipitated sample (cropped immunoblot image), (**B**) Fold change of proteins eNOS and Hcy, (**C**) Fold change of Cav-1, (**D**) Representative images of DAF-2DA fluorescence in MGECs treated without and with Hcy and H_2_S, and (**E**) Bar graph showing mean fluorescence ± SEM of DAF-2DA in MGECs. Magnification × 60, scale bar: 20 µm. MW: Molecular weight. n = 5/group, *p < 0.05 vs. CBS+/− mice without H_2_S treatment, ^†^p < 0.05 vs. WT groups, *in vitro* experiments, *p < 0.05 vs. Hcy, ^†^p < 0.05 vs. Ctr and H_2_S.

In order to determine whether the protein-amino acid (eNOS-homocysteine) interaction and thus homocysteinylation of eNOS occurred, coimmunoprecipitation experiment was done. In CBS+/− mice, there was a prominent interaction of Hcy with eNOS as observed by eNOS immunoprecipitation followed by Hcy immunodetection which showed high levels of Hcy and low levels of eNOS (Fig. 4A,B). In CBS+/− mice which received H_2_S, there was significant reduction of Hcy and increased eNOS expression (Fig. 4A,B). In the WT mice without or with H_2_S, Hcy and eNOS levels were not affected by the treatment (Fig. 4A,B).

In the non-immunoprecipitated samples, CBS+/− mice demonstrated increased caveolin-1 expression, which reduced to normal upon H_2_S supplementation (Fig. 4A,C). Caveolin-1 expression was similar in WT groups (Fig. 4A,C).

Normally upon stimulus, endothelial cells produce NO by the activation of eNOS, which diffuses into the smooth muscle cells to further activate soluble guanylyl cyclase resulting in vessel relaxation. We therefore checked for NO production in MGECs by challenging it with acetylcholine as a measure of eNOS activity. MGECs treated with Hcy only, did not increase NO generation via eNOS stimulation as indicated by low fluorescence with DAF-2DA (Fig. 4D,E). In contrast, acetylcholine enhanced the fluorescence of MGECs treated with Hcy + H_2_S (Fig. 4D,E). There was no difference in fluorescence in MGECs to H_2_S treatment alone, which was similar to the control group (Fig. 4D).

### H_2_S antagonizes ECM protein accumulation and smooth muscle cell proliferation

The activation of matrix metalloproteinases (MMPs) and inhibition of their inhibitors, tissue inhibitors of metalloproteinases (TIMPs), lead to excess collagen deposition and allows for vascular smooth muscle cell proliferation in the vasculature.

**Figure 5:**
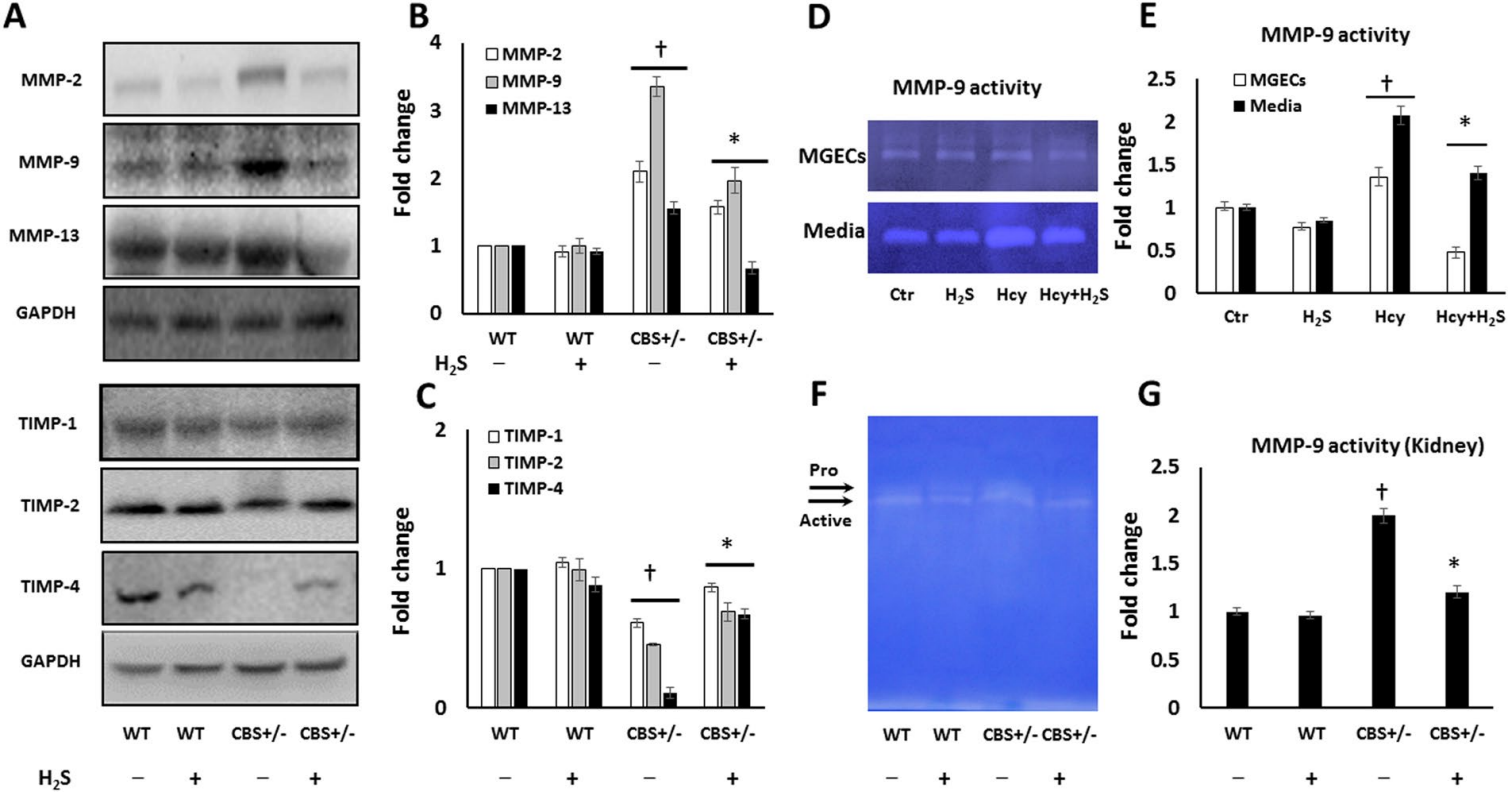
The expressions of MMP/TIMP is altered during Hhcy and H_2_S restores normal MMP/TIMP balance and reduces collagen deposition. (**A**) Representative cropped immunoblot images of protein expression of MMP-2, -9 and -13 (MW: 72, 82, and 45 kDa resp.) and TIMP-1, -2 and -4 (MW: 23, 21 and 26 kDa resp.); (**B,C**) MMP and TIMP fold change, (**D**,**E**) Representative gelatin zymogram in MGECs and media and bar graph, (**F**,**G**) Zymography of kidney lysate and bar graph. Analysis was done using ImageJ. Values are expressed as fold change. MW: Molecular weight. n = 6/group for Western blot and n = 4/group for zymography, *p < 0.05 vs. CBS+/− mice without H_2_S treatment, ^†^p < 0.05 vs. WT groups, *in* *vitro* experiments, *p < 0.05 vs. Hcy, ^†^p < 0.05 vs. Ctr and H_2_S.

MMP-2 and -9 are gelatinases which cleave denatured collagen and collagen IV in the basement membrane and MMP-13 degrades fibrillar collagen. In CBS+/− mice, MMP-2, -9, and -13 were upregulated compared to the other groups (Fig. 5A,B). Further, CBS+/− mice demonstrated significant decrease in TIMP-1 and -2 compared to WT groups (Fig. 5A,C). TIMP-4 expression was nonexistent in CBS+/− mice compared to WT groups (Fig. 5A,C). H_2_S treatment to CBS+/− mice reduced the expression of MMP-2, -9, and -13 and upregulated TIMP-1, -2 and -4 (Fig. 5A–C). In the WT groups, the expression of MMP-2, -9 and -13 and TIMP-1 and -2 was similar without or with H_2_S treatment (Fig. 5A–C). There was a non-significant decrease in TIMP-4 in WT mice treated with H_2_S (Fig. 5A,C).

MMP-9 activity was determined in *in vitro* experiments using MGECs and lysate from the kidney by gelatin zymography. Hcy treated cell lysate and media showed 1.3 and 2.03-fold increase in MMP-9 activity respectively compared to cells that did not receive any treatment (Fig. 5D,E). H_2_S treatment reduced MMP-9 activity significantly in cells treated with Hcy but did not affect cells treated with H_2_S only (Fig. 5D,E). Similarly, kidney lysate from CBS+/− mice showed nearly 2-fold increase in MMP-9 activity compared to WT mice (Fig. 5F,G). MMP-9 activity was mitigated by H_2_S supplementation (Fig. 5F,G). There was no difference in MMP-9 activity in WT groups (Fig. 5F,G).

**Figure 6:**
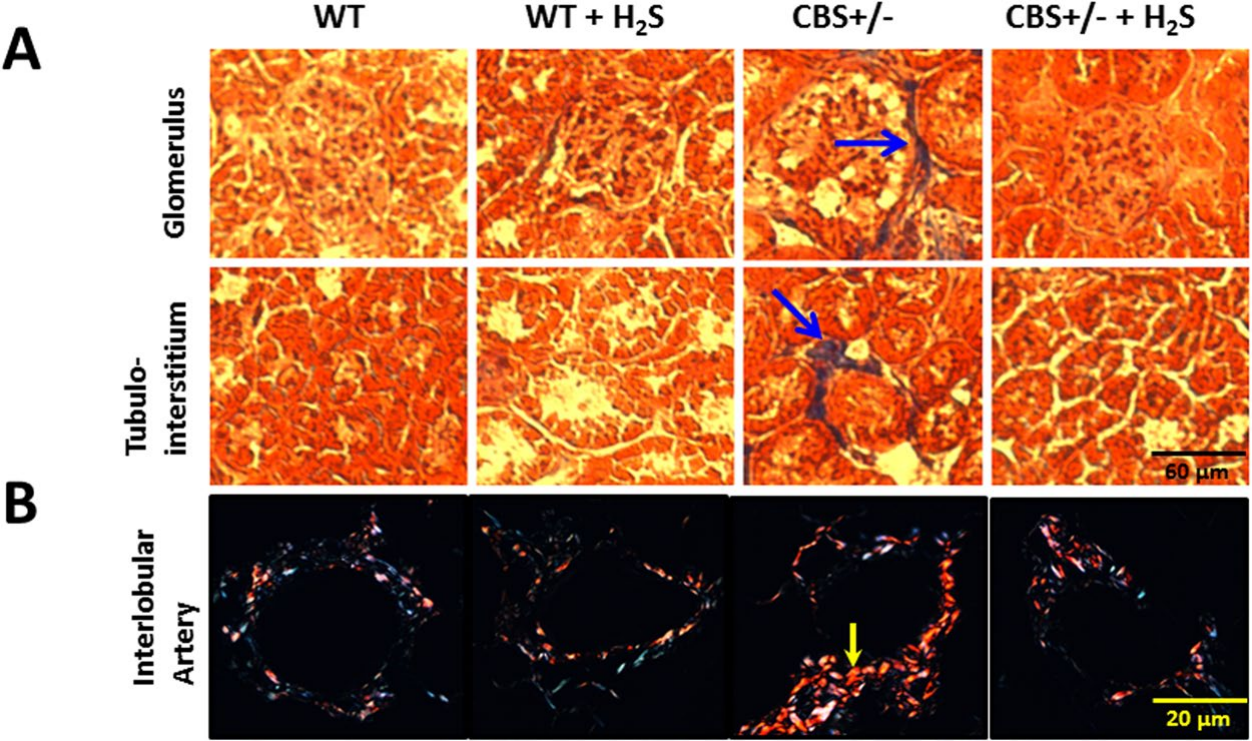
Hhcy increased collagen deposition in the glomeruli, tubulointerstitium and renal cortical arteries. (**A**) Representative images of Masson Trichrome staining for collagen (blue arrows), (**B**) Interlobular arteries show increased collagen type I (yellow arrow) with pricrosirius red stain. Magnification ×20 for Masson Trichrome, scale bar: 60 µm, and magnification ×100 for picrosirius red stain, scale bar: 20 µm. n = 5, *p < 0.05 vs. CBS+/− mice without H_2_S treatment, ^†^p < 0.05 vs. WT groups.

Renal fibrosis can occur in all compartments of the kidney. In CBS+/− mice, Masson Trichrome staining revealed increased collagen accumulation in the glomeruli and tubulointerstitium (Fig. 6A, blue arrows). In CBS+/− kidney sections stained with picrosirius red, there was increased type I collagen in the interlobular arteries suggesting arteriosclerosis (Fig. 6B, yellow arrow). Collagen deposition was reduced in all areas following H_2_S supplementation (Fig. 6A,B). WT mice treated with H_2_S had similar collagen as that of WT control mice (Fig. 6A,B).

**Figure 7:**
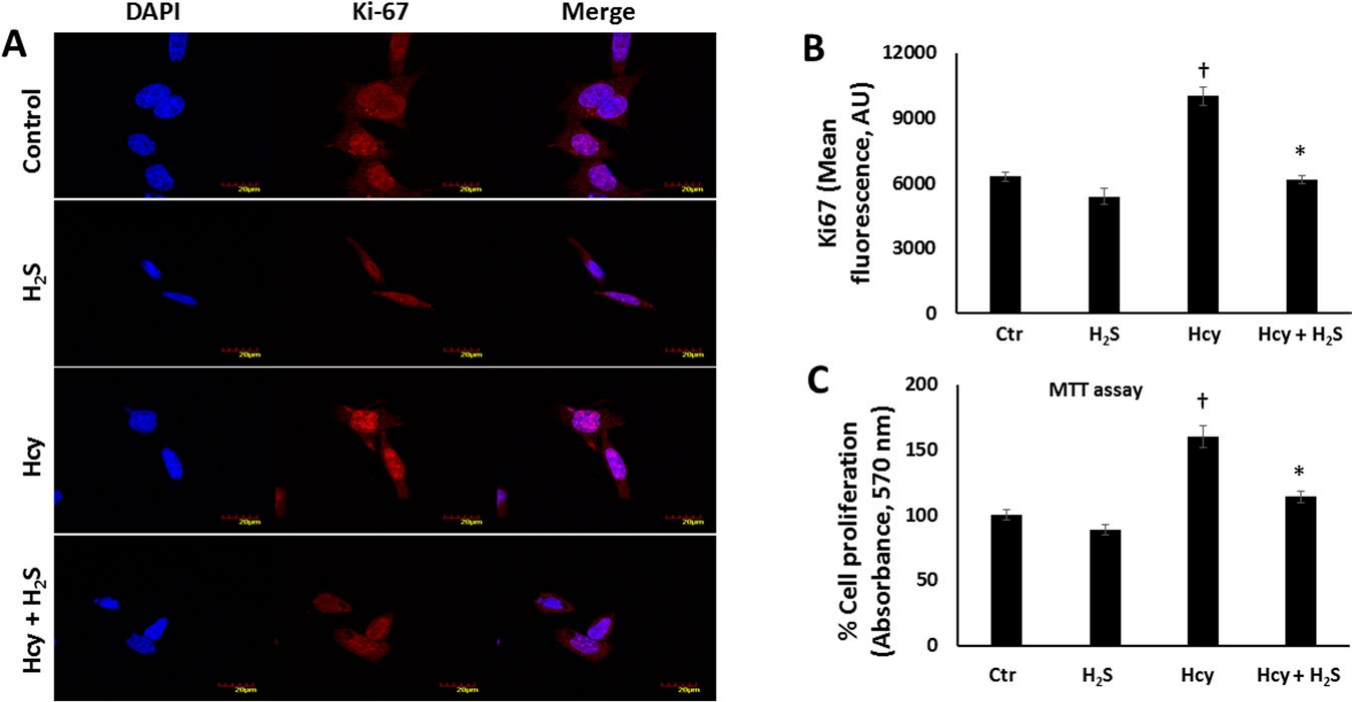
H_2_S reduces Hhcy-induced vascular smooth muscle cell proliferation. (**A**) Fluorescence images of Ki-67 in VSMCs treated without or with Hcy (75 µM) and H_2_S (30 µM), (**B**) Quantification of Ki-67, C) MTT assay showing relative VSMCs proliferation to control over 48 h. Magnification ×60, scale bar: 20 µm. n = 4, *p < 0.05 vs. Hcy, ^†^p < 0.05 vs. Ctr and H_2_S.

In separate experiments, we examined for vascular smooth cell proliferation (VSMCs) using Ki-67 marker. VSMCs treated with Hcy (75 µM) showed increased Ki-67 expression in the nucleus and decreased following H_2_S treatment (Fig. 7A,B). Since increased cell proliferation corresponds to increased metabolic activity, we performed MTT assay to confirm the findings above. There was increased absorbance in the VSMCs treated with Hcy (75 µM) alone compared to VSMCs without or with H_2_S (Fig. 7C) indicating increased metabolic activity. VSMCs treated with Hcy and H_2_S showed significant reduction in metabolism which was comparable to untreated VSMCs (Fig. 7C).

**Figure 8:**
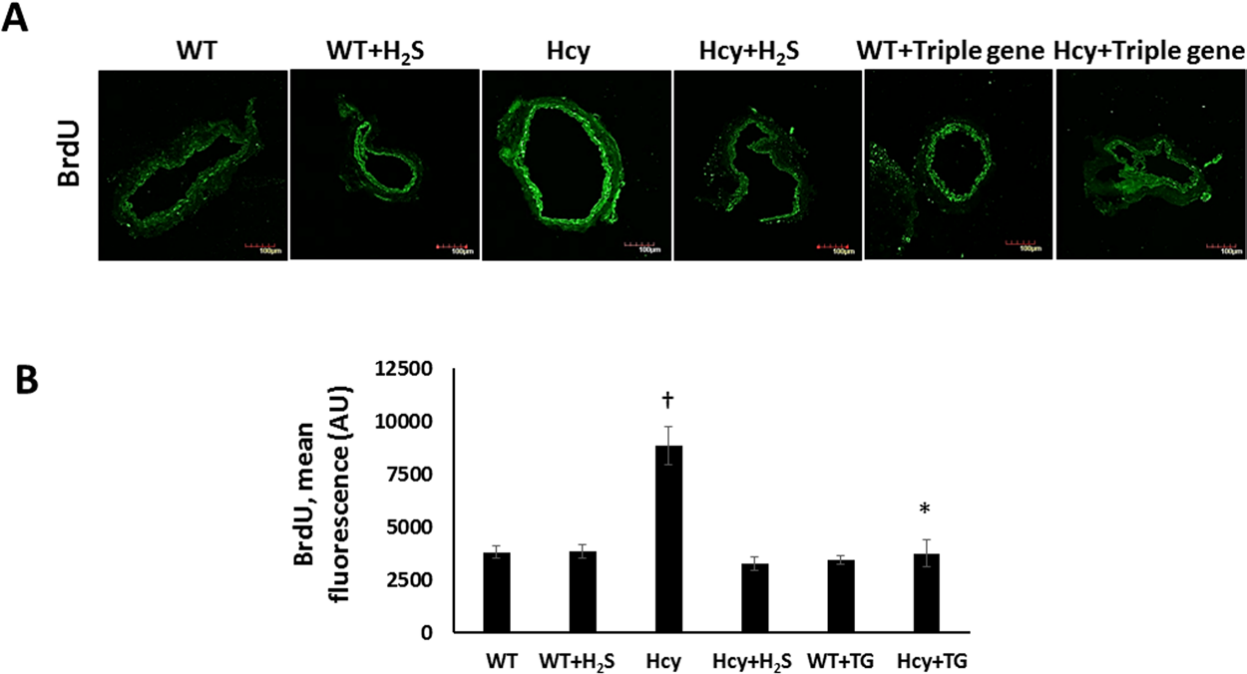
Triple gene transfection reduces BrdU incorporation in Hhcy mice. Fluorescence image of renal arteries. (**A**) Triple gene transfection (CBS, CSE and 3MST) and H_2_S reduces BrdU incorporation in renal artery explants in Hhcy, (**B**) The number of strong fluorescence signals representing BrdU were analyzed using ImageJ software. Renal artery triple gene transfection: n = 4/group, magnification ×100, scale bar: 100 µm. *p < 0.05 vs. Hcy, ^†^p < 0.05 vs. other groups.

The thymidine analog, 5′-bromo-2′-deoxyuridine (BrdU), is incorporated into newly synthesized DNA of replicating cells. Because H_2_S treatment reduced cell proliferation in Hcy treated VSMCs above, we wanted to test whether H_2_S treatment to renal artery explants would inhibit VSMCs proliferation in the tunica media. Renal arteries treated with Hcy alone showed increased BrdU immunostaining (Fig. 8A,B). In response to H_2_S, the arteries that were treated with Hcy showed significant reduction in BrdU fluorescence (Fig. 8A,B) suggesting reduction in VSMCs proliferation.

To confirm whether enhancing the endogenous activity of H_2_S producing enzymes (CBS, CSE and 3MST) would similarly affect VSMCs proliferation in renal arteries, we transfected CBS, CSE and 3MST enzymes *ex vivo* into renal arteries of WT mice (Fig. 8A,B). As expected, triple gene transfection reduced BrdU incorporation in renal artery rings of WT mice that was treated with Hcy, which was similar to H_2_S treated WT artery explants (Fig. 8A). There was no change in the expression of BrdU to triple gene transfection in WT artery explant (Fig. 8A,B).

### Hhcy increases connexin expression

**Figure 9:**
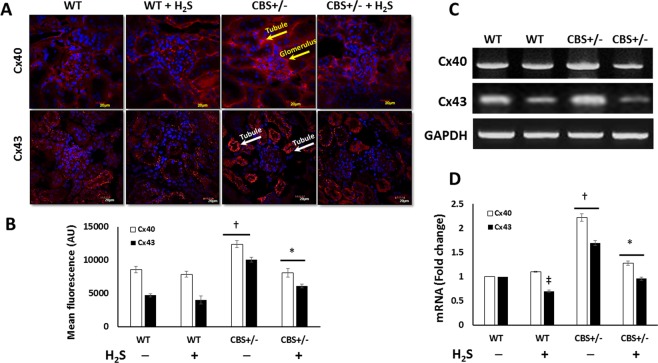
H_2_S downregulates Hhcy-induced increased expression of connexin 40 and 43. (**A**) Representative images of Cx40 in glomeruli and tubular regions (yellow arrows) and Cx43 in the tubules (white arrows), (**B**) Mean fluorescence analysis, (**C,D**) mRNA expression and fold change. Quantification was done using ImageJ software. Magnification × 60, scale bar: 20 µm, n = 6/group, *p < 0.05 vs. CBS+/− mice without H_2_S treatment, ^†^p < 0.05 vs. WT groups, ^‡^p < 0.05 vs. WT (control).

In addition to intercellular communication, connexins have multiple other roles such as cell signaling, mesangial cell proliferation, blood pressure regulation and as mediators of interstitial fibrosis. We therefore checked for changes in the expression of connexin40 and 43 (Cx40 and 43 respectively), as they are widely expressed in the kidney. In CBS+/− mice, both Cx40 and 43 was highly expressed (Fig. 9A,B). Cx40 was seen in the glomerular and tubular regions (Fig. 9A,B, yellow arrows) and Cx43 localized predominantly to the tubules (Fig. 9A,B, white arrows). H_2_S supplementation reduced the expression of Cx40 and 43 in CBS+/− mice similar to that seen in WT groups (Fig. 9A,B). H_2_S treatment did not affect the expression of connexins in WT mice. mRNA quantification for Cx40 and 43 confirmed the findings above (Fig. 9C,D).

## Discussion

This study demonstrates that Hhcy reduces hydrogen sulfide (H_2_S) generation in the kidney and homocysteinylation of eNOS and upregulation caveolin-1 expression together reduce eNOS activity resulting in hypertension. Hhcy associated renal vascular changes include reduction in vascular density, blood flow and increased smooth muscle cell proliferation contributing to impaired renal function. Further, Hhcy-induced MMP-2, -9 and -13 activation, decreased TIMP-1, -2 and -4 and upregulation of gap junction Cx40 and 43 led to dysregulation of ECM metabolism and excess collagen deposition in the glomerular and interstitial regions. We found that H_2_S supplementation dehomocysteinylates eNOS and reduces caveolin-1 expression to increase eNOS activity thus decreasing blood pressure. In addition, H_2_S restored normal MMPs/TIMPs axis to reduce glomerular and tubulointerstial sclerosis and improved renal function.

Heterozygous CBS+/− mice have approximately 50% reduction in the CBS enzyme activity and twice the normal plasma Hcy levels, which is clinically significant. Although there has been much debate whether Hhcy contributes to the development of hypertension, emerging evidence suggests it has a significant role. Earlier, a case control study demonstrated that an increase in 5 µM of Hcy was associated with an increase of systolic blood pressure by 0.7 and 1.2 mm of Hg in men and women respectively^3^. More recently, Yang *et al*. demonstrated that high levels of homocysteine is associated with hypertension in the presence of comorbidities such as, obesity, dyslipidemia and family h/o of hypertension suggesting a complex relationship between Hhcy and the development of hypertension^21^. In CKD patients, Hcy and cysteine levels are consistently increased which are also the principal substrates for H_2_S generation in the body^22,23^. However, during Hhcy, the serum levels of H_2_S was found to be significantly decreased in CKD patients^24^. Several studies have shown that H_2_S controls vascular tone and reduces blood pressure^25,26^. Conversely, low levels of H_2_S has been observed in the kidneys of spontaneously hypertensive rats and after targeted deletion of CSE leading to hypertension^25,27^. The findings from the present study adds further credence that Hhcy and low levels of H_2_S contribute to hypertension and renal pathology and supplementation of H_2_S reduces systolic blood pressure. Further, exogenous H_2_S supplementation is reported to increase CSE activity, which may therefore reduce plasma Hcy levels as observed in our study^28^.

Several studies have documented that Hhcy impairs endothelial function in various tissues^29–31^. In renal failure, endothelial and kidney production of NO is impaired^32^. As a result, there is loss of vascular relaxation, which over time leads to microvascular disruption, decreased blood flow and hypoxia. Capillary rarefaction is a crucial stage in the development of renal damage and fibrosis^33,34^. In the present study, we observed a reduction in the renal vascularity during Hhcy that was associated with reduction of cortical blood flow and poor renal function. H_2_S is a well-known vasodilator and direct infusion into the renal artery was shown to increase renal blood flow, GFR, and urinary excretion of sodium and potassium^35^. Exogenous supplementation of H_2_S in this study showed increase in the terminal branches of the renal cortex suggesting improved perfusion.

The vascular tone is regulated by the constriction and relaxation of vascular smooth muscle cells which is in part regulated by nitric oxide (NO)^36^. The endothelium-derived eNOS catalyzes the formation of NO from L-arginine. A decrease in the expression/function of eNOS can therefore affect NO production and thus impair vascular function. Protein homocysteinylation is one of the mechanisms proposed that contributes to Hhcy induced pathology in the kidney^37^. Further, homocysteinylation of plasma proteins has been documented in uremic patients undergoing hemodialysis^38^. Several other studies reported homocysteinylation of plasma proteins including albumin, hemoglobin, fibrinogen, and others such as, E-Cadherin, and actin during Hhcy and have been associated with multiple diseases^39,40^. Since endothelial dysfunction is a feature of Hhcy, we queried whether eNOS was a target of Hhcy. Our findings show that during Hhcy, eNOS is homocysteinylated and its activity is diminished in the kidney.

The eNOS activity in the endothelial cells is regulated by its interaction with caveolin-1. In an *in vitro* study on human coronary artery endothelial cells, Hhcy was found to downregulate caveolin-1 expression and translocation of eNOS from the caveolar fraction to non-caveolar fractions in the cytoplasm thereby decreasing eNOS availability^41^. However, in other studies of acute renal insufficiency, a marked increase in caveolin-1 expression was observed in the kidney including aorta and liver^42,43^. The increased caveolin-1 expression seen in the present study during Hhcy are in agreement with the latter studies above as it also explains decreased eNOS availability due to its binding to eNOS.

Nitric oxide and H_2_S have vasoregulatory roles in the body and a significant cross talk exists between the two molecules^44^. In a recent study, Wesseling *et al*. demonstrated that the gasotransmitters NO, H_2_S and carbon monoxide (CO) have a complex relationship in the development of hypertension and renal injury^45^. A reduction of H_2_S was associated with reduction of NO products but enhanced CO and CO appears to be a mediator between NO and H_2_S molecules^45^. In another study, H_2_S was shown to increase eNOS phosphorylation by activating PI3K/Akt pathway to increase NO production^44^. In animal models of left ventricle hypertrophy and acute myocardial injury, exogenous H_2_S increased NO by upregulating eNOS activity to offer cardiac protection^46,47^. In the present study, exogenous supplementation enhanced eNOS expression and activity in Hhcy and decreased caveolin-1 expression suggesting increased NO bioavailability. NO has been shown to increase CSE expression, thus H_2_S, in mouse aortic endothelial cells of caveolin1−/− mice to offer protection against atherosclerosis^48^. The decrease in caveolin-1 expression seen in our study could therefore be due to negative regulation of caveolin-1 by H_2_S or caveolin-1 translocation from the membrane into the cytoplasm for degradation to enable increased eNOS activation. These possible mechanisms need further exploration.

The disruption of MMP/TIMP axis and excess accumulation of ECM proteins during Hhcy has been demonstrated in several studies including our own^49–51^. The gelatinases, MMP-2 and -9, have increased affinity to collagen IV in the basement membrane. Their inhibitors, TIMPs, are known to have dual role. For example, at low concentration TIMP-2 activates MMP-2 whereas high concentration inhibits MMP-2^52^. TIMP-1 is the main inhibitor of MMP-9 and TIMP-4 has low affinity for MMP-9^53,54^. Therefore, low levels of TIMP-1 and TIMP-4 can upregulate MMP-9 as seen in the present study. Although the gelatinases degrade collagen, because collagen turnover is faster than elastin, oxidatively modified collagen is deposited in the ECM^55^. In this study, the upregulation of MMPs and collagen deposition led to global fibrosis in the kidney.

An essential component of vascular fibrosis involves VSMC proliferation leading to vessel stiffness. Hhcy promotes VSMCs proliferation in a ROS dependent manner and the use of antioxidant/Hcy lowering agent such as, folic acid, has been shown to inhibit proliferation^56–58^. Further, H_2_S treatment has been shown to inhibit VSMCs in several *in vitro* and *in vivo* studies^59–61^. Zhong *et al*. demonstrated that exogenous H_2_S treatment reversed VSMCs proliferation in diabetic rat mesentery^62^. Du *et al*. showed that H_2_S decreased the incorporation of [^3^H]-thymidine, a cell proliferation marker, in rat aortic VSMCs^59^. Our *in vitro* data supports these earlier findings, further, we confirmed inhibition of VSMCs proliferation in the renal artery explants by triple gene transfection which showed decreased BrdU immunofluorescence.

Connexins are gap junction proteins that play a prominent role in maintaining intercellular communication and cell homeostasis. A disruption in their expression can result from oxidative stress and inflammation both of which are associated with Hhcy. Cx40 upregulation was observed in 2 kidney, 1 clip, rat model of hypertension^63^. Cx43 showed increased expression in the damaged tubular areas of patients suffering from glomerulonephritis^64^. In another study involving rat model of puromycin-induced nephritis, Cx43 was upregulated in glomerular podocytes however, whether this increase worsens renal injury or is beneficial was not known^65^. The present study showed similar increase in Cx43 expression in the tubular areas and in the glomeruli of Hhcy mice and was associated with renal damage. Previously, we demonstrated that reduction of H_2_S in diabetic mice was associated with NMDA-R1 and Cx40 and 43 mediated renal fibrosis^66^. In the present study, H_2_S reduced the expression of Cx40 and 43 to mitigate renal injury.

## Conclusion

In this study, we show that during Hhcy, homocysteinylation of eNOS and upregulation of caveolin-1 results in decreased eNOS and NO production and a concurrent decrease in H_2_S leads to impaired vasomotor response, hypertension and poor renal perfusion. In addition, Hhcy disrupts the MMP/TIMP balance and increased Cx40 and 43 expression leading to adverse ECM remodeling. Exogenous H_2_S supplementation and triple gene therapy (CBS/CSE/3MST) dehomocysteinylated eNOS and reduced caveolin-1 to increase eNOS availability. Finally, H_2_S inhibited renovascular fibrosis and improved renal function in Hhcy-induced renal injury.

## Materials and Methods

C57BL/6J (wild type, WT) and B6.129P2-*Cbs*^*tm1unc*^/J (CBS+/−) mice on WT background were purchased from Jackson Laboratory (Bar Harbor, ME). Heterozygous CBS (CBS+/−) mice represent hyperhomocysteinemia (HHcy) model and mice aged 8–12 weeks, weighing 25 g approx. were used in this study. All animals were housed in rooms regulated in temperature (24 °C), 12:12 h light-dark cycle with access to standard chow. All handling of animals were performed in accordance with the guidelines of the animal care and use committee of the Louisville School of Medicine and the Guide for the Care and Use of Laboratory Animals published by NIH, 2011. All the experiments in the study were approved by the Institutional Animal Care and Use Committee (IACUC) at University of Louisville.

WT and CBS+/− mice (n = 7/group) received sodium hydrogen sulfide (NaHS, 30 µM), a H_2_S donor, in drinking water and their respective controls received plain water for 8 weeks. Because NaHS has short half-life, the water containing NaHS was changed at 3-h interval during the day.

### Antibodies and reagents

Sodium hydrogen sulfide (NaHS) was purchased from Sigma Aldrich (St. Louis, MO), eNOS antibody from BD Biosciences (Cat. no.: 610297, San Jose, CA), anti-homocysteine (Cat. no.: AB15154), caveolin-1 (Cat. no.: PA5–37506), connexin-40 (Cat. no.: AB38580) and Connexin-43 (Cat. no.: Ab11370) and Ki-67 (AB15580) antibodies from Abcam (Cambridge, MA), BrdU, MMP-2-9 and -13 (Cat. nos.: MAB4072, AB19167, AB19016 and AB8120 respectively) from Millipore (Burlington, MA), TIMP-1, -2, -4 and GAPDH (Cat. nos.: SC5538, SC6835, SC9375 and SC47724 resp.) and secondary antibodies for mouse and rabbit (SC358920 and SC2004 resp.) from Santa Cruz Biotechnology (Dallas, TX), Masson Trichrome kit from Fisher Scientific, USA, Picrosirius from Polysciences Inc, (Warrington, PA) and MTT assay kit from Cayman Chemical (Ann Arbor, MI).

### Blood pressure measurement by DSI telemetry

BP was measured continuously in conscious mice using PhysioTel telemetry system (DSI telemetry, St. Paul, MN) as described previously^67^. Under tribromoethanol anesthesia, the pressure transmitter, TA11PA-C10, was surgically introduced via the right carotid artery into the aortic arch. After a one-week recovery period, individual mice in cages were placed on receivers, which captured digital signals and relayed it to a computer via DSI matrix. Data was viewed and analyzed by Ponemah v5.20 software (DSI).

### High Performance Liquid Chromatography

Plasma homocysteine levels were measured by HPLC as described previously^51^. Briefly, the following were added to a microcentrifuge tube: 200 µl of plasma, 100 µl of water, 300 µl of 9 M urea (pH 9.0), 50 µl of n-amyl alcohol, and 50 µl 10% NaBH_4_ solution (wt/vol in 0.1 N NaOH). The solution was incubated at 50 °C for 30 minutes. After cooling to room temperature, 500 µl of 20% trichloroacetic acid was added to precipitate protein. Samples were centrifuged at 12,000 g for 5 minutes. and the supernatant was collected after passing through 0.22 µm filter. The sample was used for HPLC analysis. The mobile phase solution was a mixture of 0.1 M monochloroacetic acid and 1.8 mM octylsulfate in HPLC grade water at pH 3.2. The constant flow rate of isocratic solvent was 0.8 ml/minute. A Shimadzu Class-VP 5.0 chromatograph (Shimadzu, Columbia, MD) system was used to analyze samples.

### Plasma H_2_S measurement

Plasma H_2_S levels were measured as described before^68^. Briefly, freshly collected plasma (100 µL) was mixed with PBS (350 µL) and Zinc acetate (250 µL) and sealed immediately. To this mixture, N,N-dimethyl-p-phenylenediamine sulfate (20 mM, 133 μL) in 7.2 M HCl, and FeCl3 (30 mM, 133 μL) in 1.2 M HCl was added and incubated at 37 °C for 45 minutes. The reaction was terminated by adding Trichloroacetic acid solution (10% w/v, 250 μL). After centrifugation (×2,700 g for 5 minutes), aliquots were transferred to a 96-well plate and absorbance was read at 670 nm using Spectramax M2e (Molecular devices, CA). The values were calculated by using a standard curve of known concentrations.

### Laser Doppler flowmetry and Barium angiography

Renal cortical blood flow was measured using Speckle Contrast Imager (MoorFLPI, Wilmington, DE) as described before^69^. The Moor FLPI speckle contrast imager uses a diverging infrared laser beam to illuminate the tissue of interest to produce an interference pattern called as ‘laser speckle’. A charge couple device sensor in the camera captures the light and the inbuilt software processes the high and low contrast areas to yield video and trace lines. The flux units are automatically calculated by the software as No. of RBCs × velocity. The left kidney was exposed posteriorly and positioned approximately 15 cm from the camera which was focused on the kidney, renal vessels and aorta. High-speed images with a display rate of 25 Hz including line traces were obtained for 2 minutes.

Renal angiography was done by infusing Barium sulfate solution in 50 mM of TRIS buffer (100 mg/mL at pH 5.0) through the renal artery and imaged using KODAK *in vivo* Imaging System FX Pro (Molecular Imaging System; www.Bruker.com)^69^. Analysis was done using Vessel Segmentation and Analysis software (http://www.isip.uni-luebeck.de/?id=150)^69^.

### Glomerular filtration rate (GFR) measurement

GFR was measured by FITC-Inulin clearance method^70^. Mini osmotic pumps (Alzet Model 2001D, Cupertino, CA) were loaded with 200 µL of dialysed 5% FITC-Inulin. Under 2,2,2-Tribromoethanol anesthesia, osmotic pumps were implanted in the abdominal cavity. After 2 h recovery, the mice were placed individually in metabolic cages and urine was collected over 24 h period including blood samples. Since pH affects the FITC fluorescence, urine and plasma samples were buffered to pH 7.4 with 500 mM HEPES. Separate standard curves were prepared for urine and plasma as described before^71^. Samples were transferred to 96-well plate and fluorescence measured using SpectraMax M2^e^ (San Jose, CA) with excitation set at 485 nm and emission at 538 nm. Inulin clearance (GFR) was calculated as urinary FITC-Inulin excretion rate (Fluorescence counts/24 h) divided by plasma FITC-Inulin concentration (Fluorescence counts/µL).

### Immunoprecipitation analysis

Protein was extracted from whole kidney lysate and pre cleared with 20 μL of Protein A/G Plus-Agarose beads and 1 μg mouse IgG (Cat. no. SC2003, and SC2025 resp., Santa Cruz Biotechnology, Dallas, TX) at 4 °C for 30 minutes. The pre cleared lysate was incubated overnight with IgG_1_, κ eNOS mouse monoclonal antibody (2 μg/200 μg of protein, Cat. no.: 610297, BD Biosciences, San Jose, CA) at 4 °C with rotation. Protein A/G agarose beads were then added to the lysate and incubated for 4 h at 4 °C under rotation to capture the immune complex. After washing with lysis buffer, the immune complex was eluted at 50 °C for 10 minutes using SDS buffer and subjected to Western blot analysis with anti-eNOS (Cat. no.: 610297, dil.: 1:2500, BD Biosciences) and anti Hcy antibodies (Cat. no.: AB15154, dil.: 1:1000, Abcam). Secondary antibodies (rabbit anti-mouse IgG-HRP, Cat. no.: SC358920; and goat anti-rabbit IgG-HRP, Cat. no.: SC2004 respectively) were used at 1:1000 dilution and bands were detected using chemiluminescence (Luminata Forte, BioRad). Band intensity was calculated using imageJ software (https://imagej.nih.gov/ij/).

### Histology

Formalin fixed, paraffin embedded kidney sections (5 µm thickness) were stained for collagen using Masson Trichrome kit (Thermo Scientific) and Picrosirius red kit (Polysciences, Warrington, PA) following manufacturer’s instructions. Images were captured using Olympus Fluoview1000 microscope (B&B Microscope Ltd, PA).

### Western blotting

The effect of HHcy and H_2_S on the expression of eNOS, Caveolin-1, MMP-2, -9, -13 (Dilution: 1:1000 for all) and TIMP-1, -2 and -4 (Dilution: 1:300, 1:500 and 1:500 resp.) were quantified by standard Western blot^72^.

### Immunohistochemistry

Immunostaining was done for connexin-40 and -43, using 5 μm thickness frozen kidney sections. Briefly, after fixation with 4% paraformaldehyde, sections were blocked for 45 minutes at room temperature and incubated overnight at 4 °C with primary antibodies for Cx-40 and -43 (dilution: 1:100). Goat anti-rabbit IgG secondary antibody, Alexa Fluor 594 (Cat. no.:A-11012, dilution: 1:500) was applied for 90 minutes at room temperature. Images were captured by Olympus FluoView1000 (B&B Microscope Ltd., PA). Mean fluorescent intensity was quantified using ImageJ software (https://imagej.nih.gov/ij/).

### Nitric oxide production

4,5-Diaminofluorescein diacetate (DAF-2DA) was used to measure nitric oxide levels in *in vitro* experiments. Mouse glomerular endothelial cells (MGECs) were purchased from Cell Biologics (Chicago, IL) and cultured using endothelial cell media (M1168-kit, Cell Biologics, Chicago, IL). For imaging study, cells were cultured in 8-well chamber slides (Nunc, ThermoFisher) to yield a density of 3 × 10^3^ cells/well. Cells were treated without or with H_2_S (30 µM) followed by Hcy (75 µM). After 48 h, the cells were washed in PBS and incubated with 10 µM of DAF-2DA in a humidified chamber for 15 minutes at 37 °C. Subsequently, cells were challenged with acetylcholine (10^−5^ M) for 15 minutes. The cells were washed in PBS and images captured by fluorescence microscope (Olympus FluoView1000 (B&B Microscope Ltd., PA) set for excitation at 495 nm and emission at 515 nm.

### Cell proliferation studies

3-(4 – 5-methylthiozol-2 – yl)-2.5-diphenyltetrazolium bromide (MTT) assay was done using mouse aortic vascular smooth muscle cells (VSMCs, ATCC, Manassas, VA). Cells were cultured in 96-well culture plates and treated without or with H_2_S (30 μM) followed by Hcy (75 μM) for 48 h. After washing, wells were incubated with 5 mg/mL (12 mM) of MTT for 4 h at 37 °C. The formazan crystals formed were solubilized by adding solution containing 10% SDS in 0.01 M HCL and incubation for 4 h at 37 °C. Absorbance was read at 570 nm using microplate reader (SpectraMax M2e, Molecular devices, San Jose, CA).

Ki-67 is a nuclear marker for proliferating cells. In a second set of experiments, VSMCs were treated with Hcy and H_2_S as above and stained with anti-Ki-67.

In separate experiments, renal arteries from WT mice were transfected *ex vivo* with triple gene (CBS, CSE and 3MST) as described before^73^. Renal arteries were excised from WT mice (n = 4) and rinsed in ice-cold physiological salt solution. The arteries were cut into 2 mm rings and placed in a 12-well plate containing DMEM F/12 50/50 cell culture media (Mediatech Inc, VA). Arteries were transfected with plasmid containing CBS, CSE and 3MST genes (0.4 μg DNA/cm^2^ of growth area) or control plasmid without genes using jetPRIME transfection reagent (Polyplus Transfection) following the manufacturer’s instructions. After 6 h, the arteries were mounted on the bottom of plate using Matrigel (BD Biosciences, San Diego, CA) and treated without or with Hcy (75 µM) in fresh growth media. After 48 h, artery rings were fixed with Tissue Tek OCT compound. Sections of 5 μm thickness were cut and stained with 5-bromo-2′-deoxyuridine (BrdU) antibody (Millipore Sigma, MA) and images were captured with Olympus FluoView1000 microscope (B&B Microscope Ltd., PA).

### Gelatin Zymography

The proteolytic activity of MMP-9 to HHcy and H_2_S treatment was detected by gelatin zymography as described previously^69^.

### Statistics

Values are presented as mean ± SEM. Data was analyzed by ANOVA followed by post hoc, Bonferroni correction to identify differences between two groups. Mann-Whitney U test was done for nonparametric data. A ‘p’ value of <0.05 was considered significant.

## Supplementary information

Supplementary information
