# Supplementary material for: Hydrogen Sulfide Protects Hyperhomocysteinemia-Induced Renal Damage by Modulation of Caveolin and eNOS Interaction
Source: Sci Rep. 2019 Feb 18;9:2223. doi: 10.1038/s41598-018-38467-6 (PMC6379383; doi:10.1038/s41598-018-38467-6)
Supplement: Supplementary file 1 — Supplementary information [file 41598_2018_38467_MOESM1_ESM.docx]

Supplementary information

**Hydrogen Sulfide Protects Hyperhomocysteinemia-Induced Renal Damage by Modulation of Caveolin and eNOS Interaction**

Sathnur Pushpakumar^1^, MD, PhD, Sourav Kundu^2^, PhD, Utpal Sen^1^, PhD

^1^Department of Physiology, School of Medicine, University of Louisville, Louisville, KY 40292

^2^Department of Botany, West Bengal State University, Berunanpukria, Kolkata, West Bengal, India, PIN 700126

Running title: Caveolin and eNOS modulation in Hyperhomocysteinemia

Key words: Hyperhomocysteinemia, Caveolin, eNOS, Hydrogen sulfide

Word count: 5132

Figures: 9 (total)

Correspondence:

Utpal Sen, Ph.D.

Associate Professor

Dept. of Physiology

500 S Preston St.

HSC-A, Room 1115,

University of Louisville,

KY-40202,

Ph: 502-852-2030;

Fax: 502-852-6239;

Email: [utpal.sen@louisville.edu](mailto:utpal.sen@louisville.edu)

Figure 4A


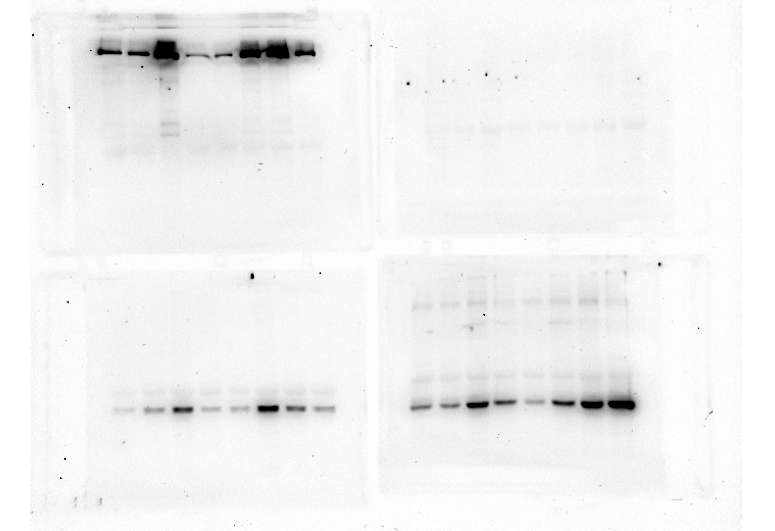


**20 kDa**

**Cav-1**

Supplementary figure S1: Uncropped image for Caveolin-1 (Cav-1) expression.

Figure 5 A


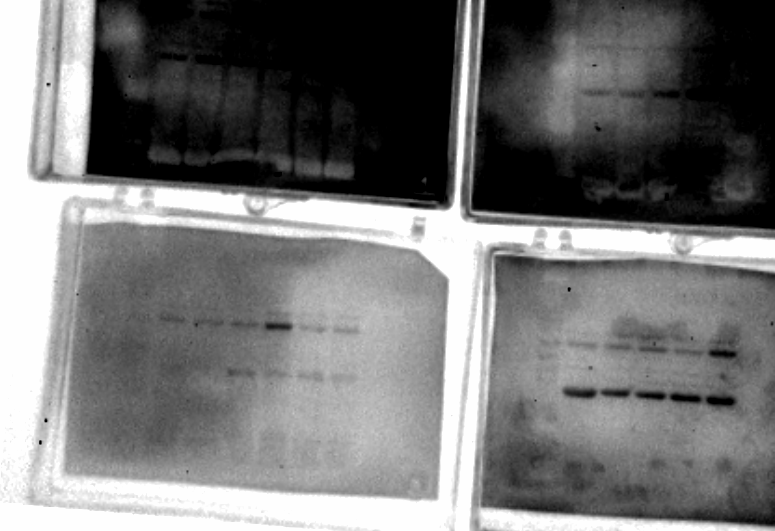


**72 kDa**

**MMP-2**

**MMP-9**

**82 kDa**


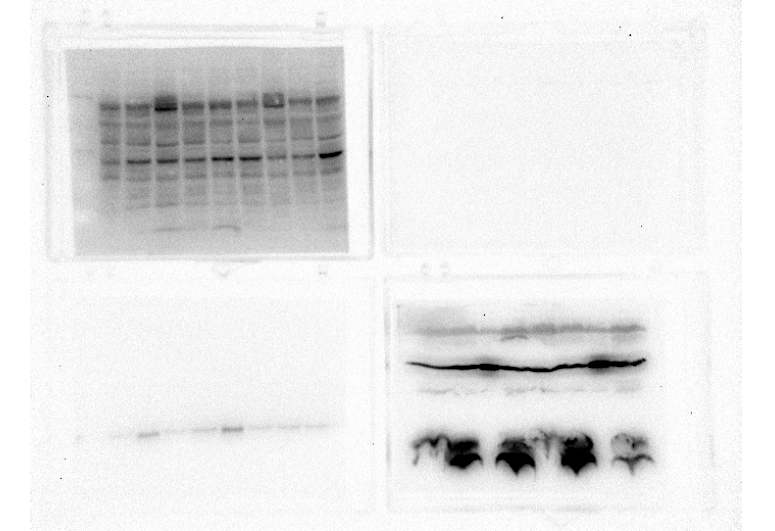

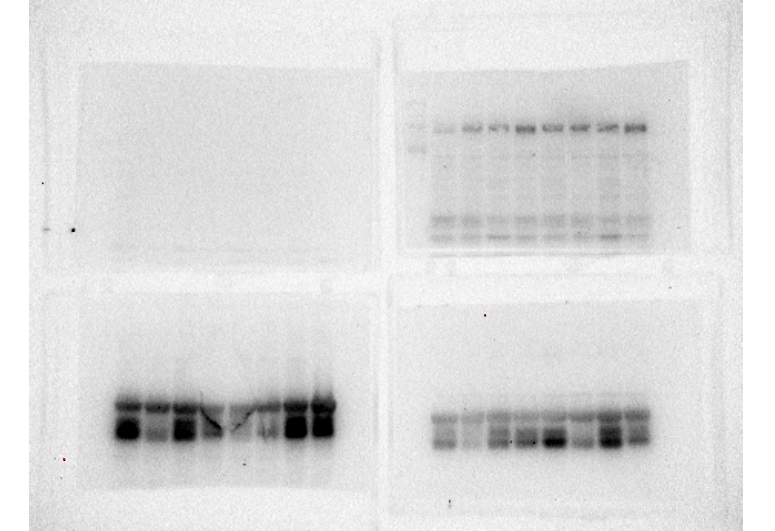


**MMP-13**

**45 kDa**

Supplementary figure S2: Uncropped images for MMP-2, -9 and -13 expression.

Figure 5 A


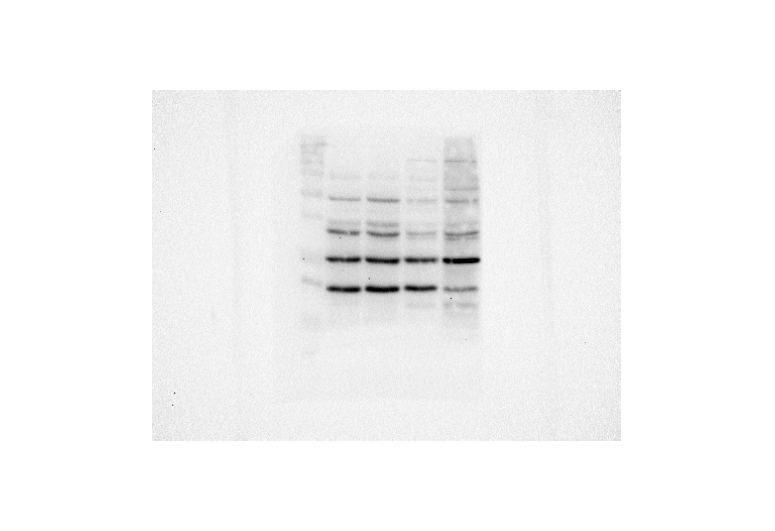


**TIMP-1**

**23 kDa**

**TIMP-2**

**21 kDa**


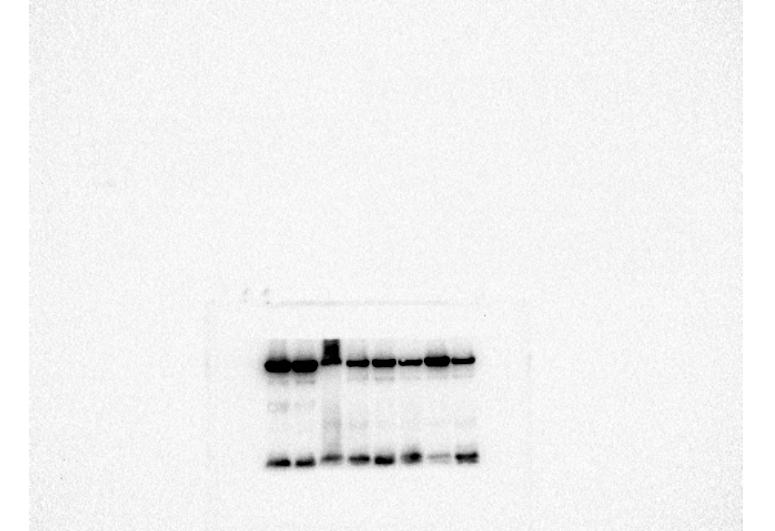

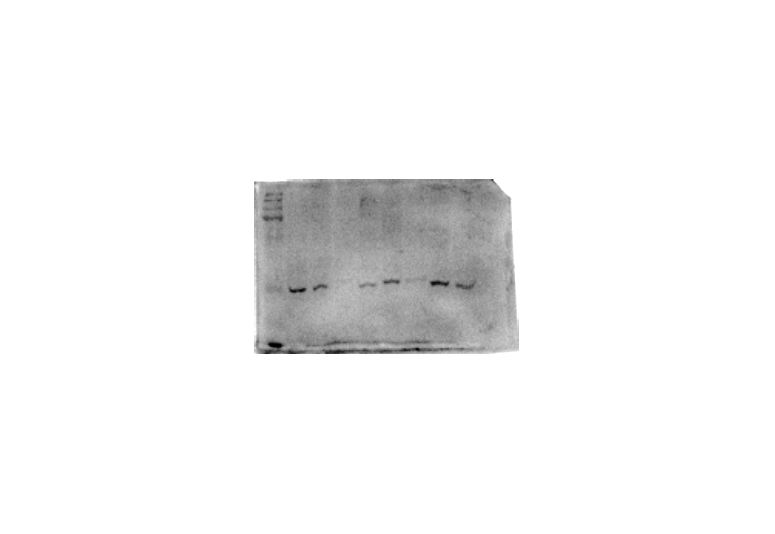


**TIMP-4**

**26 kDa**

Supplementary figure S3: Uncropped images for TIMP-1, -2, and -4 expression.
